# Retrospective analysis of single-center experience in ultrasound-guided puncture drainage for pediatric appendiceal abscess

**DOI:** 10.3389/fped.2026.1802899

**Published:** 2026-05-14

**Authors:** Chuankai Lv, Zhiru Wang, Cheng Zhang, Xin Wei, Xiaoman Wang

**Affiliations:** 1 Department of Ultrasonic, Beijing Children’s Hospital, Capital Medical University, National Center for Children’s Health, Beijing, China; 2 Department of Emergency Surgery, Beijing Children’s Hospital, Capital Medical University, National Center for Children’s Health, Beijing, China

**Keywords:** appendiceal abscess, local anesthesia, pediatric, prognosis, ultrasound-guided puncture drainage

## Abstract

**Objective:**

To evaluate the clinical efficacy of ultrasound-guided puncture drainage for pediatric appendiceal abscess and summarize operational experience.

**Methods:**

This study conducted a retrospective analysis of clinical data from pediatric patients who underwent ultrasound-guided percutaneous drainage for appendiceal abscess at Beijing Children's Hospital between January 2022 and June 2025. The safety and efficacy of ultrasound-guided abscess drainage were analyzed, along with key technical points and experiences for different types of appendiceal abscesses. Categorical variables were compared using the *χ*^2^ or Fisher's exact test, and continuous variables were analyzed using the *t*-test or Mann–Whitney *U* test. A *P*-value < 0.05 was considered statistically significant.

**Results:**

A total of 90 children underwent ultrasound-guided puncture of the appendiceal abscess and were included in this study, and the control group consisted of 41 children with appendiceal abscess who received conservative treatment during the same period. Children who underwent ultrasound-guided abscess drainage under local anesthesia showed a faster decrease in white blood cell count (7.0 ± 2.0 vs. 11.0 ± 3.0 × 10^9^/L; *t* = 4.0, *P* < 0.05) and C-reactive protein levels (7.3 ± 2.1 vs. 11.3 ± 3.1 mg/L; *t* = −7.6, *P* < 0.05) after one week of treatment, shorter duration of abdominal pain (4.3 ± 1.5 vs. 5.8 ± 1.3 days; *t* = −5.7, *P* < 0.05), and quicker resolution of the abscess compared (11.3 ± 1.2 vs. 15.7 ± 1.1 days; *t* = −21.1, *P* < 0.05) to those who received intravenous antibiotics alone. Ultrasound-guided abscess drainage resulted in minimal trauma and lower rates of early and late postoperative complications. A retrospective review of ultrasound data from 90 children who underwent ultrasound-guided abscess drainage demonstrated that our hospital had extensive operational experience in performing drainage for various types of complicated appendiceal abscesses.

**Conclusion:**

Ultrasound-guided percutaneous drainage is a safe and effective minimally invasive treatment for pediatric appendiceal abscess in children who can cooperate with local anesthesia. Physicians must master the indications for drainage and specific puncture techniques for different types of appendiceal abscesses.

## 1 Introduction

Appendiceal abscess is one of the common acute abdominal diseases in children, characterized by the accumulation of pus resulting from a suppurative or perforated appendix attached to the surrounding tissues. The greater omentum and intestines wrap around and adhere to the appendix, forming an inflammatory mass. For appendiceal abscesses with a prolonged history, surgical intervention becomes challenging due to severe adhesions, making appendectomy difficult and resulting in significant surgical trauma. For pediatric patients with established appendiceal abscess, ultrasound-guided abscess puncture and drainage offers advantages including minimal invasiveness, absence of radiation exposure, and no requirement for general anesthesia. However, due to the unique physiological characteristics of the pediatric population and the relatively high procedural complexity, prior ultrasound-guided puncture procedures have largely depended on individual clinicians’ experience, frequently utilizing static ultrasound localization (i.e., puncture at a predetermined site identified by ultrasound) rather than real-time ultrasound guidance. Existing literature predominantly addresses the necessity of drainage, optimal timing of intervention, or post-drainage management strategies (such as interval appendectomy), with limited focus on the technical specifics of the puncture technique itself (1).

This study aimed to analyze the safety and efficacy of ultrasound-guided percutaneous drainage of appendiceal abscesses under local anesthesia in pediatric patients through a retrospective study. Additionally, it sought to summarize the operational experience of ultrasound-guided aspiration for different types of appendiceal abscesses and promote its application.

## 2 Materials and methods

### 2.1 Study population

This study retrospectively analyzed the clinical data of pediatric patients diagnosed with appendiceal abscess at Beijing Children's Hospital from January 2022 to June 2025, who underwent either ultrasound-guided percutaneous drainage of the appendiceal abscess or received antibiotic therapy alone. The cohort included 90 cases of ultrasound-guided drainage procedures. The study protocol was approved by the hospital's ethics committee, and written informed consent was obtained from all participants’ parents or legal guardians.

The inclusion criteria were: 1) Age ≤ 18 years; 2) Confirmed diagnosis of appendiceal abscess with no surgical indication (Patients with a history of abdominal pain lasting for more than 5 days or even 7 days, without diffuse peritonitis or septic shock); 3) Complete medical records, drainage procedure documentation, and follow-up data; 4) Follow-up duration of at least three months.

The exclusion criteria were: 1) Post-appendectomy abscess; 2) Presence of other intra-abdominal infection sources; 3) Incomplete treatment at our hospital; 4) Incomplete medical records. Criteria for determining pediatric patients suitable for antibiotic conservative therapy alone without percutaneous abscess drainage: 1) Ultrasound evaluation reveals no safe needle trajectory for puncture due to obstruction by adjacent structures (e.g., bowel, blood vessels, bladder, mesentery). 2) The child is too young or has poor cooperation, rendering local anesthesia unfeasible. 3) The abscess is small and diffusely distributed, or the pus within the cavity is dense/thick, making aspiration technically difficult with anticipated poor procedural efficacy. 4) Presence of coagulopathy or other underlying conditions that increase the risk of invasive procedures (e.g., hemophilia). 5) Parental refusal of puncture procedure.

### 2.2 Instruments and operating procedures

All children who underwent ultrasound-guided appendiceal abscess drainage or conservative treatment were assessed by experienced clinicians and deemed not suitable for immediate surgical intervention. The ultrasound-guided appendiceal abscess drainage procedures were performed by experienced sonographers using the Hitachi-vision Ascendus (Hitachi, Tokyo, Japan) and DIT-DD70 (DIT, Wuhan, China) ultrasound machine, with 16G/18G needles (Leapmed, Jiangsu, China) selected for the procedure. During operation, the frequency of the abdominal ultrasound convex probe is (3.5–5.0) MHz, the frequency of the L12-5 high-frequency probe is (5–12) MHz, and the frequency of the L7-3 medium-frequency probe is (3–7) MHz. All patients received the procedure under local anesthesia.

Puncture procedure: Prior to the procedure, perform an abdominal ultrasound scan to locate an appropriate puncture site. Position the child in a supine position (or in a knee-chest or lithotomy position if a perineal approach is required), followed by routine disinfection and draping. Under ultrasound guidance, administer 5 mL of 1% lidocaine for layered infiltration anesthesia from the puncture site down to the peritoneal layer, ensuring adequate anesthesia to avoid pain during the puncture. Under real-time ultrasound guidance, insert a 16/18G needle into the deep or mid-lower portion of the appendiceal abscess, slowly aspirate the pus, and adjust the needle position as needed. The needle can be removed once the abscess cavity is significantly reduced and no further pus can be aspirated. After the puncture, perform a full abdominal ultrasound scan to confirm the absence of bleeding or collateral damage, followed by sterile local pressure dressing.

All children who underwent ultrasound-guided percutaneous aspiration of appendiceal abscess had bacterial culture performed on the aspirated pus. Regardless of whether drainage is performed, children with appendiceal abscess require full-course and adequate antibiotic therapy. The antibiotic regimens for both groups consist of cefoperazone-sulbactam combined with metronidazole or ertapenem for anti-infective treatment. If positive microbiological results are obtained during treatment, adjust the antibiotics according to the susceptibility results.

### 2.3 Observation indicators

The collected data included patient age, gender, weight, changes in laboratory parameters before and after one week of treatment, time to resolution of clinical symptoms, duration of antibiotic use, etc. Additionally, data were collected on the duration of the puncture procedure, ultrasound imaging information, early postoperative complications (such as bleeding, intestinal perforation, etc.), and long-term complications (such as intestinal obstruction, etc.) in children who underwent ultrasound-guided puncture and drainage therapy.

### 2.4 Statistical analysis

Data processing was performed using SPSS 27.0 statistical software. All measurement data underwent normality tests and homogeneity of variance tests. Normally distributed measurement data were expressed as mean ± standard deviation (Mean ± SD), and comparisons between two independent sample groups were conducted using independent samples *t*-test or *t*'-test based on whether the variance was homogeneous. Non-normally distributed measurement data were expressed as [M (P25, P75)], and intergroup comparisons were performed using the Mann–Whitney *U* test. Categorical data were presented as frequencies and percentages [n (%)], and intergroup comparisons were conducted using chi-square (*χ*²) test or Fisher's exact test. A *P*-value < 0.05 was considered statistically significant.

## 3 Results

### 3.1 General characteristics of the two groups

The gender composition showed no significant differences between the puncture and non-puncture groups. Children in the ultrasound-guided percutaneous puncture group for appendiceal abscess were older in age (*t* = 2.4, *P* = 0.02) and had greater body weight (*Z* = 2.1, *P* = 0.03) compared to those in the non puncture group. This suggests that age was an important consideration when assessing whether pediatric patients were suitable for ultrasound-guided local anesthesia during appendiceal abscess puncture, as better patient cooperation was required. Additionally, the evaluation should incorporate abscess size (*t* = 6.1, *P* < 0.05), as ultrasound-guided puncture may be feasible for children with larger abscess volumes. Details were presented in Table 1.

**Table 1:** Comparison of general characteristics between the puncture and non puncture groups.

Group	Cases (n)	Male (n)	Female (n)	Age (months)	Weight (kg)	The longest diameter of the abscess (mm）
Puncture	90	44	46	85.5 ± 27.5	34.6 (14.9–52.9)	57.8 ± 12.2
Non Puncture	41	20	21	54.3 ± 36.7	26.5 (13.2–49.7)	43.5 ± 10.3
Statistic	-	0.0002^a^		2.4^b^	2.1^c^	6.1^b^
*P*-value	-	>0.05		0.02*	0.03*	< 0.05

a The chi-square (*χ*^2^) test was used for frequency data.

b Parametric quantitative data are presented as mean ± standard deviation (SD) and were analyzed using the *t*-test.

c Non-parametric quantitative data are presented as median (P25–P75) and were analyzed using the Mann–Whitney *U* test.

*A *P*-value < 0.05 was considered statistically significant.

### 3.2 Comparison of the main outcomes between the two groups

The statistical analysis was conducted with the evaluation of early treatment efficacy in children at the 1-week post-treatment time point. This study found no significant differences in white blood cell count (WBC) and C-reactive protein (CRP) levels between the two groups of children before treatment. However, one week after treatment, the reduction in WBC (*t* = 4.0, *P* < 0.05) and CRP (*t* = −7.6, *P* < 0.05) levels was significantly greater in the ultrasound-guided abscess puncture group compared to the non-puncture group. When analyzing the time to resolution of clinical symptoms in children as an outcome measure, this study found that the time to disappearance of abdominal pain symptoms was significantly shorter in children who underwent ultrasound-guided abscess puncture compared to the non-puncture group (*t* = −5.7, *P* < 0.05). In some cases, abdominal pain was markedly relieved on the same day as the puncture procedure. However, there was no significant difference between the two groups in the time to resolution of fever symptoms (*t* = −1.6, *P* = 0.11). Additionally, it was observed that the resolution of intra-abdominal abscesses was faster in children who received ultrasound-guided puncture (*t* = −21.1, *P* < 0.05), even when they initially had larger abscesses. Details are presented in Table 2.

**Table 2:** Evaluation of treatment efficacy: comparison of changes in infection-related parameters and time to resolution of clinical symptoms in pediatric patients between the puncture and non puncture groups.

Observed Indicator		Puncture	Non Puncture	*t*	*P*-value
WBC (×10^9^/L)	Before treatment	17.5 ± 2.9	16.4 ± 2.7	2.1	0.04*
	After treatment	7.0 ± 2.0	11.0 ± 3.0	4.0	<0.05*
CRP (mg/L)	Before treatment	93.3 ± 23.2	91.3 ± 21.2	0.5	0.3
	After treatment	7.3 ± 2.1	11.3 ± 3.1	−7.6	<0.05*
resolution of abdominal pain (d)		4.3 ± 1.5	5.8 ± 1.3	−5.7	<0.05*
recovery time of body temperature (d)		3.2 ± 0.8	3.5 ± 1.1	−1.6	0.1
resolution of abscess (d)		11.3 ± 1.2	15.7 ± 1.1	−21.1	<0.05*

*A *P*-value < 0.05 was considered statistically significant.

### 3.3 Complications and long-term prognostic impact

Conduct statistical analysis on early and late complications as well as long-term survival outcomes of all children who underwent ultrasound-guided percutaneous drainage of appendiceal abscess to verify its safety. Among the 90 children, one developed intra-abdominal hemorrhage within 24 h post-procedure (hemoglobin decreased by 14 g/L), which stabilized after symptomatic hemostatic treatment. No cases of intestinal perforation caused by the procedure occurred. Four children experienced varying degrees of intestinal obstruction symptoms during treatment, which were attributed to adhesions between intestinal loops caused by the appendiceal abscess itself. All 90 children achieved abscess resolution after ultrasound-guided percutaneous drainage combined with a full course of adequate antibiotic therapy. Among them, 31 children underwent elective laparoscopic appendectomy 3 months after cure, with an average operative time of 65.0 (46.5, 107.5) minutes, and no cases required conversion to open surgery.

### 3.4 The ultrasound manifestations and typical features of the appendiceal abscesses

**Figure 1:**
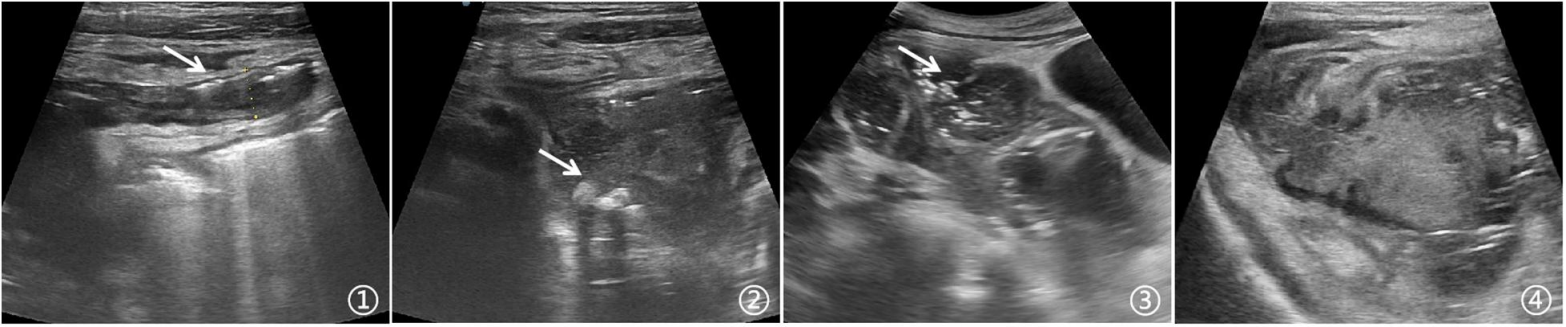
**(1)** The ultrasound can visualize the appendix with a roughly displayed appearance, and this interface shows the long-axis view of the appendix. The outer diameter of the appendix measures 0.97 cm. **(2)** A free appendicolith is visible within the abscess cavity. **(3)** A multi-loculated appendiceal abscess with multiple punctate gas echoes inside. **(4)** The abscess cavity is encapsulated, and the pus inside has a viscous consistency.

The sonographic manifestations of appendiceal abscess were diverse. In most cases, the complete or partial structure of the appendix could be identified on ultrasound, with the abscess either encasing the appendix or closely associated with it. The appendix typically exhibited a widened external diameter, indistinct layers of the appendiceal wall, and swelling of the surrounding mesentery and intestinal wall. Scattered appendicoliths in the abdominal cavity might also be observed, serving as crucial evidence to confirm the abscess's origin from the appendix. When gas entered the abdominal cavity from the perforation site of the appendix or when gas-producing bacteria were present, punctate hyperechoic gas reflections might be seen within the abscess cavity. Appendiceal abscesses usually presented as thick yellow or yellowish-green pus, and ultrasound guidance could roughly assess the viscosity of the pus, thereby aiding in the selection of the appropriate needle size and predicting the difficulty of the puncture procedure. Typical images were illustrated as shown in Figure 1.

### 3.5 Selection of puncture points for appendiceal abscesses at different locations

**Figure 2:**
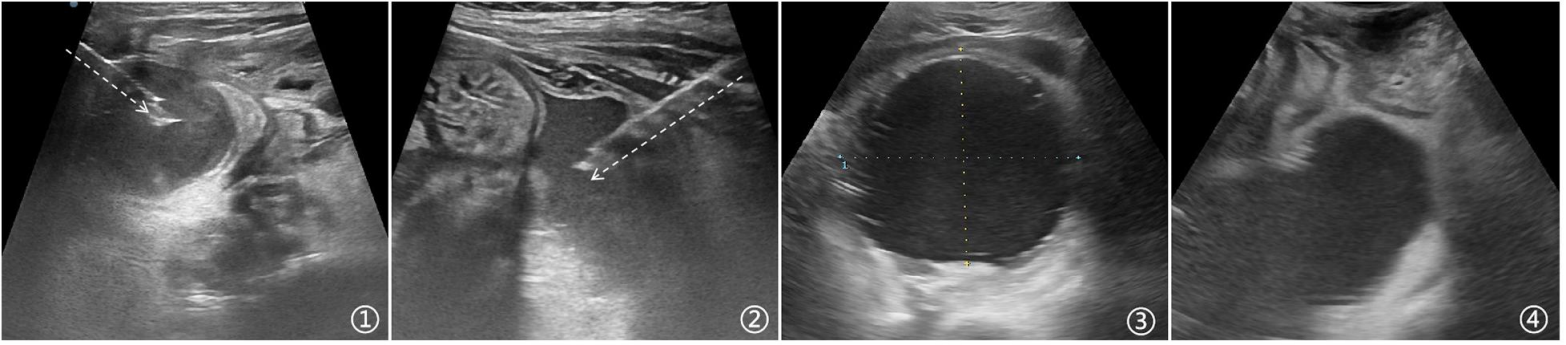
Appendiceal abscesses at different locations and the selection of puncture sites. **(1)** A single abscess cavity around the appendix in the right lower abdomen can be drained by inserting a needle for pus aspiration from the right lower abdomen, with the needle path visible along the arrow direction. **(2)** If the abscess is located in the left lower abdomen, ultrasound can be used to locate and select an appropriate entry point in the left lower abdomen for needle insertion. **(3)** The abscess is located behind the bladder, and under ultrasound, the bladder appears non-distended while the abscess is relatively large. **(4)** For an abscess located anterior to the rectum, this type of appendiceal abscess can be punctured via the perineal approach.

Appendiceal abscesses were located in various positions within the abdominal cavity, and the choice of needle insertion points for drainage varied depending on the abscess location. The most common site for appendiceal abscesses was the right lower quadrant, often extending downward into the pelvis, making the area around McBurney's point the most frequently used puncture site. In some children, the appendix was positioned closer to the midline or in the left abdomen, resulting in abscesses concentrated on the left side of the abdominal cavity. In such cases, real-time ultrasound guidance was used to select an appropriate puncture point on the left side. When the appendiceal abscess primarily accumulated in the pelvis, the puncture point was adjusted downward, and needle insertion was performed from either the left or right side above the bladder, with care taken to avoid damaging critical structures such as the bladder, uterus, or ovaries. If the abscess was located deep in the pelvis and no suitable abdominal puncture point was accessible, the child was positioned in the knee-chest or lithotomy position, allowing for transrectal or perineal drainage of the appendiceal abscess. Typical images were illustrated as shown in Figure 2.

### 3.6 Technical points of ultrasound-guided puncture for complex appendiceal abscesses

**Figure 3:**
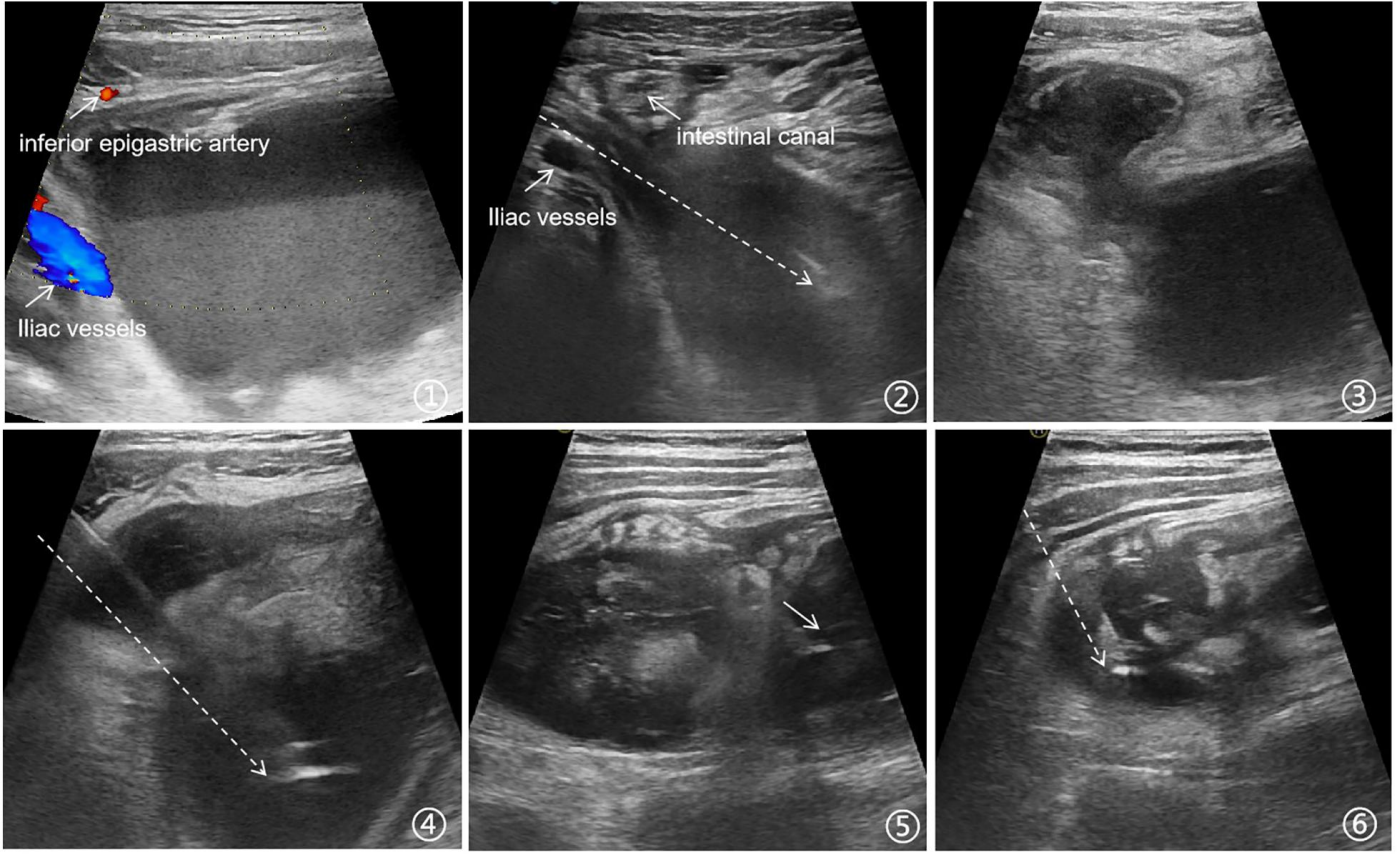
Management of complicated appendiceal abscess. **(1)** The appendiceal abscess is large and appears close to the inferior epigastric artery and iliac vessels. **(2)** Under ultrasound guidance, the needle is advanced while avoiding critical structures such as the iliac vessels and intestinal canal. **(3)** A longitudinally arranged, tandem series of appendiceal abscesses with communication between the two abscess cavities. **(4)** From the same patient as in Image 3, the needle traverses all abscess regions, preferentially targeting the bottom abscess for aspiration before gradually withdrawing the needle. **(5)** A transversely arranged, interconnected series of bead-like appendiceal abscesses where the needle's visibility is obscured; the arrow indicates the tip of the needle, located at the distal end of the abscess cavity. **(6)** From the same patient as in Image 5, after completing aspiration of the distal abscess, the needle is withdrawn and is now targeting the proximal abscess.

When performing ultrasound-guided puncture for complex and special types of appendiceal abscesses, the operator must possess extensive experience and technical skills. Appendiceal abscesses may sometimes be closely adjacent to blood vessels (such as the inferior epigastric artery, iliac vessels, etc.), mesentery, or intestines, increasing the difficulty of the procedure. Prior to puncture, careful ultrasound scanning is required to identify the positions of blood vessels and surrounding intestinal structures. The needle should be advanced slowly under real-time ultrasound guidance to ensure it passes through the narrow accessible pathway without damaging other critical tissues. In many cases, appendiceal abscesses are not single cavities but may contain multiple non-communicating loculations, necessitating multiple punctures. However, in some pediatric patients, the abscess cavities may be interconnected in a beaded pattern, either horizontally or vertically. For these cases, the puncture should first be guided by ultrasound to position the needle tip into the more distant abscess. Aspiration should be performed to gradually collapse the distal abscess, followed by real-time ultrasound-guided withdrawal of the needle tip toward the proximal cavity while continuing suction. This approach maximizes thorough drainage while maintaining optimal ultrasound visualization. Typical images are illustrated as shown in Figure 3.

## 4 Discussion

An appendiceal abscess is a localized collection of pus in the abdominal cavity caused by inflammation of the appendix, presenting as a discrete inflammatory mass with well-defined boundaries (2). In children with acute appendicitis, appendiceal abscesses account for 2%–10% of cases (1). A global burden of disease study revealed 4.5 million new cases of appendicitis in children aged 0–19 in 2021, with complicated appendicitis (including abscesses) constituting a significant proportion and imposing a heavier disease burden (3). A Finnish multicenter study reported that the incidence of appendiceal tumors in children with periappendicular abscesses was as high as 14.3%, significantly higher than in uncomplicated appendicitis (1.5%) (4). Early diagnosis and treatment of acute appendicitis in children are crucial for reducing the incidence of appendiceal abscesses. However, due to the physiological and developmental characteristics of adolescents and their limited ability to articulate symptoms, abscesses often form by the time they seek medical attention, necessitating safe and effective treatment methods to minimize disease-related harm.

Acute complicated appendicitis with periappendiceal abscess formation presents greater therapeutic challenges and typically requires a more comprehensive approach. Depending on the patient's condition, either conservative or surgical treatment may be chosen, but the optimal timing for intervention remains controversial (2). A 2017 systematic review analyzing mortality and complications of early versus delayed appendectomy for appendiceal abscesses did not yield definitive conclusions, though the study was limited by a small sample size of 40 (1). Generally, early-stage acute complicated appendicitis with periappendiceal abscess (duration of symptoms on of symptoms ≤ 72 h) is more suitable for surgical intervention. Conversely, late-stage acute complicated appendicitis (duration of symptoms > 72 h) or cases involving periappendiceal abscess formation are often managed conservatively, including percutaneous abscess drainage and antibiotics. This approach is primarily based on the fact that long-standing appendiceal abscesses often lead to intestinal adhesions and omental encapsulation. Surgical intervention in such cases may result in uncontrolled bleeding, intestinal injury, postoperative residual intra-abdominal infections, a higher incidence of adhesive intestinal obstruction, and even intestinal fistulas, causing greater patient suffering (5, 6).

Adequate abscess drainage is crucial for the treatment of children who cannot undergo early appendectomy. Surgical abscess clearance or catheter drainage is feasible, but the child must undergo a palliative general anesthesia procedure, which imposes a greater disease burden on both parents and the child. Therefore, how to perform abscess drainage in a more minimally invasive manner has become a key concern for clinicians, and many approaches have been attempted. Endoscopic retrograde appendicitis therapy (ERAT) for periappendiceal abscesses has been reported multiple times in recent years, but most cases are isolated reports, and no large-scale data are available (7, 8). Under endoscopic ultrasound (EUS) guidance, pelvic abscess drainage can be performed for pelvic abscesses. Li et al. (9) reported successful treatment of six appendiceal patients with an abscess using this technique, with puncture sites selected in the colon or rectum. However, it should be noted that there are no large-scale case reports on the use of these methods for treating appendiceal abscesses in children. Additionally, these approaches may carry risks of immediate or long-term intestinal perforation, necessitating active clinical monitoring (10).

Percutaneous drainage techniques (including CT or ultrasound guidance) have become a minimally invasive alternative for treating abdominopelvic abscesses, offering lower mortality and complication rates compared to traditional surgery and being more widely accessible than endoscopic ultrasound (1, 11). Initially used in adults, this technique has gradually been extended to the treatment of complicated appendicitis in children (such as appendiceal abscess or phlegmon). For this special pediatric population, the radiation exposure from CT should be considered in treatment selection, and ultrasound-guided drainage of appendiceal abscesses is less traumatic for children. Previous reports on ultrasound-guided drainage of appendiceal abscesses in children have been scarce, possibly due to challenges such as low patient cooperation, difficulties in standardizing ultrasound imaging, or sonographers performing only localization without conducting the puncture procedure. In our hospital's ultrasound department, real-time ultrasound-guided drainage of appendiceal abscesses is performed, with the same physician both guiding and performing the puncture. All procedures are completed under local anesthesia, achieving satisfactory outcomes and shortening the overall conservative treatment duration for pediatric patients.

Key lessons learned from our experience as follows: ① Pre-procedure comprehensive assessment: The child must be able to cooperate with local anesthesia and have a suitable access pathway. ② Ultrasound-guided local infiltration anesthesia: Anesthesia should extend to the peritoneal layer to minimize pain during puncture. ③ Flexible puncture site selection: The puncture site should not be limited to the right lower abdomen. ④ Real-time ultrasound guidance: The puncture needle should remain visible throughout the procedure to avoid collateral damage and ensure thorough aspiration of pus. ⑤ For complex appendiceal abscesses: Follow the principle of “distant before proximal” puncture to ensure optimal visualization and effectiveness.

This study represents a retrospective single-center analysis, inherently limited by potential selection bias and constraints in data completeness. Furthermore, influenced by real-world clinical admission patterns, an imbalance in sample size distribution was observed between the conservative management group and the puncture group, introducing potential bias into the results. In light of these limitations, our center has subsequently initiated a large-sample prospective randomized controlled trial to further validate the current findings, with the aim of elevating the level of evidence and enhancing clinical applicability.

In summary, for pediatric patients who can cooperate with local anesthesia, ultrasound-guided abscess aspiration is a safe and effective minimally invasive treatment for pediatric appendiceal abscess. Physicians must be proficient in the indications for drainage of different types of appendiceal abscesses and the specific puncture techniques. However, it is important to note that a full course of adequate antibiotic therapy for infection control is also essential, as such combined treatment can significantly shorten the recovery time for pediatric patients (12).

## Data Availability

The raw data supporting the conclusions of this article will be made available by the authors, without undue reservation.
